# Why Give My Surgical Patients Probiotics **

**DOI:** 10.3390/nu14204389

**Published:** 2022-10-19

**Authors:** Katerina Kotzampassi

**Affiliations:** Department of Surgery, Aristotle University of Thessaloniki, 54636 Thessaloniki, Greece; kakothe@yahoo.com

## 1. Introduction

Although there are various hypotheses on the health-promoting roles probiotic supplementation play—via targeting the gut microbiota and/or regulating the systemic immune and metabolic responses—the precise nature of this benefit in restitution of health following surgery remains under discussion and in doubt. However, based on the current literature, the reasons for which their use could be considered almost mandatory are their aptitude to: reduce systemic postoperative infections and surgical wound infections; enhance gut motility; alleviate postoperative pain; prevent antibiotic-induced diarrhea; and prevent anastomotic leaks.

As early as 1993, the surgeon, now Emeritus Professor, Stig Bengmark, from Lund, Sweden, began 10 years of laborious research—limited to fecal cultures, which were the laboratory capabilities of the time—to find strains capable of colonizing the human intestinal mucosa. This thorough investigation led to the identification and use, initially, of the strains *Lactobacillus plantarum* 299 and 299V against post-operative sepsis in major surgery patients, followed, in the new millennium, by the combination of a four Lactobacilli plus four fermentable fibers regimen, under the commercial name Synbiotic 2000Forte [1,2,3,4].

Today, 40 years later, we appreciate the usefulness and effectiveness of probiotics in counteracting many functional disturbances in vital organs and systems as well as in a bundle of illnesses. However, there is still considerable doubt and much questioning as to their success in relation to various surgical procedures, and it is as yet difficult to clearly define the most appropriate formula for each specific operation or complication, and, perhaps even more importantly, in relation to each individual’s microbiome.

## 2. To Reduce Surgery Related Complications

In spite of advances in surgical techniques, the optimized perioperative management with the ERAS protocol, and the improvement in perioperative care, nosocomial bacterial infections continue to represent a major clinical problem [5]. A number of clinical studies have directly correlated surgically related complications, mainly post-operative pneumonia and urinary tract infections, with the suppression of the host’s immune response, a gradual reduction in the gut mucosal barrier functionality, and changes in the microbial diversity; all these are attributable to surgical stress insult, with the loss of “health-promoting” commensal microbes and overgrowth of pathogenic bacteria, even in elective surgery patients [6,7,8,9]. As a consequence, surgically related complications remain unchanged over time, resulting in prolonged hospitalization, extended duration of antibiotic therapy, unscheduled re-admission, increased mortality rate, and finally high costs to healthcare systems [10,11,12].

The manipulation and restoration of the microbiome as a strategy to reduce dysbiosis through the perioperative administration of probiotics or synbiotics is a novel, very promising, infection prevention strategy, since it significantly reduces the risk of infectious complications, with the magnitude of this risk reduction approaching 50% [6,7,13,14].

A fairly recent meta-analysis of 35 trials was the first to exclusively investigate the effect and possible mechanism of action of pro- and synbiotics to lower the risk of SSIs [10]. After the administration of beneficial bacteria, the inflammatory CRP and IL-6 significantly decreased, as serum diamine oxidase [DAO]—produced at the tip of the small bowel villi and reflecting the mucosal integrity—increased. Short-chain fatty acids were also elevated, the butyrate being beneficial, apart from being an energy source for colonocytes, in inhibiting the expression of virulence genes, controlling the function of regulatory T cells and restricting the growth of *Pseudomonas aueroginosa*, a collagenase producer implicated in the pathogenesis of anastomotic leakage. This meta-analysis, comprising 3028 adult patients, confirmed that pro- and synbiotics significantly reduce the incidence of abdominal distention, diarrhea, pneumonia, sepsis, and urinary tract infection and, moreover, positively affect the duration of antibiotic therapy, of postoperative pyrexia, and of hospital stay (*p* < 0.05) [10].

Research into other, previously published meta-analyses based on individualized groups of surgeries, reported somewhat similar findings in the probiotic-/symbiotic-treated groups versus placebo. 

In 28 RCTs dealing with gastrointestinal surgery, fewer patients experienced post-op pneumonia [OR = 0.44; 95%CI, 0.28 to 0.68] and urinary tract infections [OR = 0.30, 95%CI, 0.16 to 0.55] [5]. Similarly, in gastrointestinal surgery [15 RCTs], the risk of post-op sepsis was reduced by 38% [RR = 0.62; 95%CI, 0.52 to 0.74] [15]. In colorectal cancer surgery [14 RCTs], fewer patients developed pneumonia [RR = 0.52, 95%CI, 0.29 to 0.95, *p* = 0.03], urinary tract infections [RR = 0.35, 95%CI, 0.19 to 0.67, *p* < 0.001], septicaemia [RR = 0.65, 95%CI, 0.55 to 0.78, *p* < 0.001], and central line infection [RR = 0.51, 95%CI, 0.27 to 0.96, *p* = 0.04] [16]. Incidents of pneumonia were also reduced in another similar meta-analysis of 13 RCTs [pooled OR = 0.56, 95% CI 0.32 to 0.98, *p* = 0.04] [17]. In 12 liver surgery studies, lower infection rates were presented [pooled RR = 0.46, 95%CI, 0.31 to 0.67] [18], while in 6 liver transplantation studies the overall infection rate [RR = 0.29, 95%CI, 0.14 to 0.60], urinary tract infections [OR = 0.14, 95%CI, 0.04 to 0.47] and the duration of antimicrobial therapy [WMD = −4.31, 95%CI, −5.41 to 0.47] were reduced, but there was no difference in pneumonia, peritonitis and cholangitis rates [19].

There are no studies dedicated entirely to elective surgery patients who remained intubated for an extended period post-op; however, there are many meta-analysis of RCTs dealing with critically ill patients and with ventilatory-induced pneumonia, to whom probiotics were given with good results in relation to placebo [20,21,22,23].

However, it is of interest to emphasize once again the non-homogeneity of the studies, in terms of the type and number of probiotic species used. Only 20 out of the 35 trials used probiotic regimes that were also used in other studies: one probiotic regime was used by six researchers; another by four; two different regimes by three; and another two by another two researchers. In each of the remaining 15 studies, different strains were administered. Despite these methodological difficulties, the beneficial effects of probiotics in reducing SSIs remains unquestionable, as has also recently been demonstrated in multi-trauma patients [24].

## 3. To Reduce Surgical Site Infections

Surgical site infections (SSIs), accounting for about 16% of the nosocomial infections, are the third most frequent health-care associated infections, followed by intensive care unit and urinary tract infections [25], and they remain the major cause of morbidity and mortality, despite improvements in infection control techniques. The pathogens responsible for SSI in particular depend mainly on the type of surgical procedure and the patient’s own microbiome, and to a lesser extent on exogenous sources [26]. According to an earlier investigation, carried out in 2009, in a purely US population, the 158 639 SSIs were estimated to be the most frequent (36.0%) infectious complication nationwide. On a per-case basis, SSI were found to be the third most costly at USD 20 785 (95% CI, USD 18 902–USD 22 667), and when ranked according to total annual cost, they contribute the most to overall costs (33.7% of the total) [27].

Bacteria are transferred either from the gut, according to the classic theory of bacterial translocation [28], or, according to the recently introduced “trojan horse” hypothesis [29], based on the observation that some pathogens, most notably *S. aureus*, can invade neutrophils at remote sites of colonization and remain viable intracellular pathogens after re-entering systemic circulation. As part of the normal immune response to surgery, these pathogen-laden neutrophils migrate to sites of traumatized tissue or implanted foreign material, where they release their infectious payload in parallel with other inflammatory mediators [29]. However, we must always consider the option that bacteria already natively present within the surgical site microbiome may undergo phenotypic switching from commensalism to virulence without any need for translocation [30,31].

The preventative use of probiotics in patients subjected to any kind of surgery or trauma has been extensive, either under a primary research protocol aiming to increase the commensal bacteria to fight pathogens and thus balance the microbiome, or as a consequent finding in patients treated with probiotics peri-operatively [8].

In a meta-analysis of 20 trials—1374 patients—probiotics/synbiotics were given for the assessment of their efficacy in reducing infection risk after abdominal surgery. Patients who received probiotics experienced a 37% reduction in the rate of SSI [RR = 0.63; 95%CI = 0.41 to 0.98] versus placebo [14]. In another meta-analysis of six RCTs with low heterogeneity [I2 = 11%], involving 653 colorectal surgery patients, the probiotics group demonstrated a significantly lower SSI rate than the placebo group [OR =0.62; 95%CI =0.39 to 0.99] [32], a finding quite similar to that in general surgery patients, although colon surgery is much less ‘clean’. A problematic group of patients is those subjected to hepatectomy, being at high risk for SSI [15.2%]. A meta-analysis of four RCTs found a significant decrease [6.3%] in the subgroup receiving probiotics [RR = 0.387, 95% CI = 0.155 to 0.970, *p* = 0.043], without statistical heterogeneity [33]. Similarly, in liver transplant patients (4 RCTs), SSIs significantly reduced from 35% to only 7% in the probiotics group [RR = 0.21, 95% CI = 0.11 to 0.41, *p* < 0.001] [34].

All the aforementioned studies and hundreds of others strongly suggest that probiotics provide considerable opportunities for counteracting wound infections, leading, in clinical practice, to a significantly lower incidence of surgical site infections, giving promising results and providing possible new alternatives or adjuvant therapies.

## 4. To Enhance Gut Motility

Gastrointestinal motility, as the core part of the accelerated recovery of patients undergoing gastrointestinal surgery, has important clinical significance and has received close attention from surgeons [35]. A delayed recovery of gastrointestinal function after surgery, namely, postoperative ileus, with the clinical manifestations of abdominal distension and pain, sometimes delayed gastric emptying and vomiting, and gastrointestinal dysmotility with delayed passing of first flatus and defecation, eventually leading to the prolongation of hospital stay and even increased morbidity [36,37].

The mechanism of postoperative ileus is rather multifactorial; it is classically attributed to the direct manipulation of the intestines during abdominal surgery, in co-operation with the skin and the peritoneal cavity opening, all comprising the neurological phase that is via adrenergic refluxes involving a spinal loop with afferent splanchnic nerves synapsing in the spinal cord, activating efferents travelling back to the gut [38].

Then, the inflammatory phase follows, with the release of a large number of inflammatory mediators, such as interleukin-6, interleukin-1, monocyte chemoattractant protein-1 and cell adhesion molecule-1, which damage intestinal muscles and further inhibit the recovery of gastrointestinal function. Last, but not least, anesthesia and the pharmacological interventions thereafter, mainly opioids often used as analgesics, have a major impact by means of activation of μ-opioid receptors, which in turn inhibit acetylcholine release from myenteric fibers [35,38,39].

The most recent meta-analysis, in 2022, based on 21 RCTs involving 1776 participants focused exclusively on the effect of peri-operatively given probiotics (or synbiotics) on the early postoperative recovery of gastrointestinal function. Compared to the control group, they reduced the incidence of abdominal distension and of postoperative ileus, and resulted in a shorter time to first flatus (MD, −0.53 days), first defecation (MD, −0.78 days), first fluid (MD, −0.29 days) and first solid diet (MD, −0.25 days), as well as a reduction in the length of postoperative hospital stay (MD, −1.43 days) [35].

Unfortunately, there are no investigative studies focusing precisely on the mechanisms by which the ingested probiotics positively affect colonic motility after abdominal surgery; however, there are many experimental studies on rodent models researching the mechanisms by which probiotics may modulate motility. Recent evidence indicates that bacterially derived microvesicles are capable of gastrointestinal epithelial paracellular transport [40,41] and modulating colonic motility: *L. reuteri* DSM-17938 microvesicles was found to increase colonic propagating contraction frequency in ex vivo mouse colon [42]. Toll-like receptors (TLR2 and TLR4) in intestinal epithelial cell membranes recognize resident intestinal lumen bacteria and initiate the intracellular signaling that modulates motility [40]. Similarly, bacterial metabolites act as neurotransmitters: short-chain fatty acids (SCFA) produced by gut bacteria, as the *B. lactis* HN019, from carbohydrate fermentation are capable of modulating colonic motility [43,44]. Some gut bacteria are also able to produce tryptophan-derived substances, which may potentially act on the motility modulating 5-HT3A ion channel, for which tryptamine is a partial agonist [45]. More specifically, *L. plantarum* PS128, orally given, was found to significantly increase the small intestinal transit rate in naïve mice; after 14 days of treatment, *L. plantarum* PS128 alters the expression of genes related to serotonin signal transduction, leading to an increase in the biosynthesis and storage of 5-HT in entero-chromaffin cells in the ileum, as revealed by immune-histochemical analysis. These findings suggest that *L. plantarum* PS128 promotes serotonin signal transduction in the intestine, which might indirectly affect the CNS-related functions and host behaviors through the gut–brain axis [40].

## 5. To Alleviate Postoperative Pain?

At present, there is no positive answer. However, there is much literature on the interactions of at least some probiotics and their metabolites on visceral pain and nociceptive process, functional dyspepsia and irritable bowel syndrome being the most representative manifestations of visceral pain, not excluding, however, the visceral injury after an operation. These bacteria are considered capable of synthesizing and releasing many neurotransmitters and neuromodulators, or stimulating entero-endocrine cells to synthesize and release neuropeptides and hormones.

The most well-documented research is that of Rousseaux et al. [46], who first evaluated the ability of five well-known *Lactobacilli* and *Bifidobacteria* to induce the expression of analgesic receptors. From those five, only *L. acidophilus* NCFM and *L. salivarius* Ls-33 were found to induce a sustained increase in OPRM1 mRNA (μ-opioid) expression in human HT-29 epithelial cells, and only *L. acidophilus* NCFM to induce also CNR2 mRNA (cannabinoid) expression. Then, to further validate the functional role of *L. acidophilus* NCFM-induced analgesic receptors, they used the colonic distension model: oral administration of NCFM for 15days (10^9^ cfu/d) decreased normal visceral perception, allowing a 20% increase in the pain threshold, or resulted in an anti-nociceptive effect of the same magnitude as that achieved by the subcutaneously given 0.1mg/kg morphine. The authors conclude that the direct contact of *L. acidophilus* NCFM with epithelial cells is able to induce, via the NF-κB pathway, μ-opioid and cannabinoid receptors to mediate the normal perception of visceral pain, similar to the effects of morphine.

Of course, the pain after abdominal surgery is not only due to the splanchnic injury but almost equally to the abdominal wall trauma, however small, such as after laparoscopic surgery. There are no data correlating the intensity of the inflammatory response with the severity of accelerated pain, although from the ancient times of Celsus (1st century AD) pain has been recognized as an integral to the quartet of manifestations of inflammation—redness and swelling with heat and pain.

Surgical trauma activates the immune system both directly, by the binding of danger-associated molecular patterns (DAMPs) to pattern recognition receptors of the innate immune system, and indirectly via the activation of the neuroendocrine system, through the hypothalamic–pituitary–adrenal axis, leading to the release of hormones, cytokines, chemokines, and prostanoids, which are essential to restore homeostasis and are involved in tissue repair [47]. In parallel, surgical injury leads to the activation and sensitization of the nociceptive system, through the release of different mediators, including bradykinin, prostanoids, and cytokines. Pro-inflammatory cytokines, such as TNF-α and interleukins 1β, 6, and 17, secreted at and recruited to the site of injury, have the ability to activate and increase the sensitivity to pain stimuli [48], through receptors located on the nociceptive neurons, and finally stimulate the primary afferent Aδ and C-nerve fibers and synapse with neurons in the dorsal horn of the spinal cord [49]. The neutralization of these cytokines, by any means, results in a quick reduction in pain [50].

In recent years, much research has been conducted to screen the immuno-modulatory effects of probiotics: the oral administration of *Lactobacillus* strains was found to influence the balance of Th1⁄Th2 immune response, although this effect seems to be species-, strain-, dose-, and probably time-specific [51,52]. Therefore, we have to hope that by choosing the proper combination of probiotics, a targeted reduction in pro-inflammatory cytokines will positively engage and ameliorate postoperative pain. However, generally speaking, *Lactobacillus* strains are capable of inducing pro-inflammatory cytokines such as IL-12 and IFN-γ in addition to anti-inflammatory cytokines such as IL-10 [49], whereas *Bifidobacterium* strains are generally better inducers of IL-10 than *Lactobacillus* strains [53].

## 6. To Prevent Antibiotic-Associated Diarrhea

Probiotics, even as milk-fermented products, have been used empirically for decades to alleviate the negative side effects of oral antibiotics, but understanding of the way they work is so far incomplete. A metagenomic analysis of the fecal microbiota of 135 subjects, who received a 14 d scheme of antibiotics revealed a rapid alteration of gut microbiota, a decrease in richness and diversity, a bloom of pathobionts of the family *Enterobacteriaceae*, and the depletion of several taxa including *Bifidobacterium* and butyrate producers [54]. The co-administration of probiotics showed a small, but measurable, benefit on microbiome recovery after antibiotics, which was linked to the detection and replication of specific probiotic strains.

Apart from this research, there are many meta-analyses of RCT studies reporting the positive effect of probiotics in patients who received multi-day antibiotic combinations [55,56]. The most recent one, analyzing 42 studies, adding up to 11,305 participants, reports that probiotics reduce the risk of antibiotic-associated diarrhea in adults by 37%. However, it is of interest that, in subgroup analyses, the authors underline that a high dose compared with low dose of the same probiotic demonstrated a positive protective effect; the same is true with a multi- versus single-strain regime; and only certain species, mainly of the *Lactobacillus* and *Bifidobacteria* genera, were found to be effective [56].

Unfortunately, there are no RCTs focused exclusively on the prevention of/reduction in antibiotic-associated diarrhea frequency after probiotic treatment in abdominal surgery patients. However, very few meta-analyses include such a parameter. The most recent one, focusing on probiotics/synbiotics administered perioperatively to patients undergoing colorectal cancer surgery referred to a significant reduction in diarrheal incidence [OR = 0.38, 95% CI 0.24–0.60, *p* < 0.001] in relation to the placebo group [57]; thus, probiotics treatment should be considered as having the potential to improve post-surgical gastrointestinal-related quality of life.

## 7. To Prevent Anastomotic Leaks

Anastomotic leakage remains the most devastating and lethal complication following gastrointestinal surgery, especially in the high-risk areas of the esophagus and rectum. It has a significant negative impact on disease-free survival, overall survival and local recurrence [58]. The inspired work of the Alverdy’s laboratory group for over a decade on the mechanisms of low anterior anastomosis dehiscence has finally shown that a low microbial diversity allows the overgrowth of mucin-degrading members of the *Bacteroidaceae* and *Lachnospiraceae* families. Pathogens such as *Pseudomonas aeruginosa*, *Enterococcus faecalis* and *Serratia marcescens*, with their capacity to proliferate when the microbiota become depleted, can produce collagenase and elicit intestinal inflammation, leading to anastomotic leak [59,60]. They have demonstrated in rats that anastomotic leakage occurs when *Pseudomonas aeruginosa* colonizing anastomotic sites become transformed in vivo (single-nucleotide polymorphism mutation) to express a tissue-destroying, more virulent phenotype [60] and when *Enterococcus faecalis* expresses an increased collagen-degrading activity and an increased ability to activate host tissue matrix metalloproteinase 9 (MMP9) [59]. Most recent work to verify their findings in rats has revealed that in humans the presence of collagenolytic bacteria is the most deterministic, but alone is not sufficient to cause anastomotic leakage [61]. The mechanical bowel prep, the use of oral and intravenous antibiotics, could have influenced the microbiome along with other multiple factors, including technique, blood flow and molecular elements of tissue inflammation and healing.

Seeing thus a failed anastomosis from this point of view, that is, the devastation of beneficial and increased abundance of highly pathogenic bacteria species, we can hope that the administration of probiotics could preserve/augment the number of beneficial bacteria, acting in a similar way as in the case to prevent antibiotic-associated diarrhea. Of course, there are no data at that moment to support this; however, it is well-known that probiotic bacteria are responsible for short-chain fatty acid (SCFA) production, through the anaerobic fermentation of indigestible polysaccharides, such as dietary fiber and resistant starch. The SCFAs are the key energy source for colonic cells to keep intact their tight junctions and defense against opportunistic pathogens. Thus, attempts to keep the beneficial bacteria population stable or to repopulate with the health-promoting microbiota through the use of probiotics show promise.

## 8. Conclusions—Final Thoughts

Considering all of the above, the obvious conclusion is that probiotics are now well-established for the prevention, control, limitation and treatment of surgical diseases. Certainly, their effects cannot be proven, as strong as they appear in pathological conditions. Let us not forget, however, that the studies performed in pathological conditions number some tens of thousands of cases, while in surgical diseases the meta-analyses do not exceed 5000 cases. However, the most important barrier, apart from the organism diversity between individuals, is their different reactions to surgical manipulations, which makes grouping of similar cases much more problematic—or, in other words, a very large number of cases would be required to derive reliable results.

A good motivation for the further establishment and increased use of probiotics might also be the reduction in hospitalization costs. It is well-known that huge sums are spent to deal with surgical complications and consequently increased days of hospitalization and ICU stay, while these seem to be reduced when probiotics are used. However, to date, no large cost–benefit studies have been designed to highlight the possible role of probiotics both as an excellent “alternative” or complementary therapy, but also as an intervention which reduces hospitalization costs.

Probiotics should finally be considered medicines, and not just dietary supplements, as has been the case until now. Thus, the responsibility for their validation and the checking of their content in terms of active bacteria should pass exclusively to the national drug registration council.

Given that we now recognize a multitude of microbiomes, other than the gut, our clinical research must also be directed towards the application of probiotics for the restoration of the other microbiomes, as now occurs for that of the skin. The upper respiratory tract, the oral cavity, and the anal canal are easily accessible areas, with a possible future extension to the lungs.

Finally, more research is needed: with the deep knowledge of the detailed action of each probiotic strain, combinations of probiotics could be designed to act in different, consecutive postoperative stages, preventing negative or helping towards positive disease progress.
